# NECAB3 Promotes Activation of Hypoxia-inducible factor-1 during Normoxia and Enhances Tumourigenicity of Cancer Cells

**DOI:** 10.1038/srep22784

**Published:** 2016-03-07

**Authors:** Hiroki J. Nakaoka, Toshiro Hara, Seiko Yoshino, Akane Kanamori, Yusuke Matsui, Teppei Shimamura, Hiroshi Sato, Yoshinori Murakami, Motoharu Seiki, Takeharu Sakamoto

**Affiliations:** 1 Division of Cancer Cell Research, Institute of Medical Science, The University of Tokyo, Shirokanedai, Minato-ku, Tokyo, Japan; 2 Division of Molecular Pathology, Institute of Medical Science, The University of Tokyo, Shirokanedai, Minato-ku, Tokyo, Japan; 3 Graduate School of Medicine, Nagoya University, Tsurumai-chou, Nagoya, Japan; 4 Division of Molecular Virology and Oncology, Cancer Research Institute, Kanazawa University, Kakuma-machi, Kanazawa, Japan; 5 Faculty of Medicine, Institute of Medical, Pharmaceutical and Health Sciences, Kanazawa University, Takara-machi, Kanazawa, Japan

## Abstract

Unlike most cells, cancer cells activate hypoxia inducible factor-1 (HIF-1) to use glycolysis even at normal oxygen levels, or normoxia. Therefore, HIF-1 is an attractive target in cancer therapy. However, the regulation of HIF-1 during normoxia is not well characterised, although Mint3 was recently found to activate HIF-1 in cancer cells and macrophages by suppressing the HIF-1 inhibitor, factor inhibiting HIF-1 (FIH-1). In this study, we analysed Mint3-binding proteins to investigate the mechanism by which Mint3 regulates HIF-1. Yeast two-hybrid screening using Mint3 as bait identified N-terminal EF-hand calcium binding protein 3 (NECAB3) as a novel factor regulating HIF-1 activity via Mint3. NECAB3 bound to the phosphotyrosine-binding domain of Mint3, formed a ternary complex with Mint3 and FIH-1, and co-localised with Mint3 at the Golgi apparatus. Depletion of NECAB3 decreased the expression of HIF-1 target genes and reduced glycolysis in normoxic cancer cells. NECAB3 mutants that binds Mint3 but lacks an intact monooxygenase domain also inhibited HIF-1 activation. Inhibition of NECAB3 in cancer cells by either expressing shRNAs or generating a dominant negative mutant reduced tumourigenicity. Taken together, the data indicate that NECAB3 is a promising new target for cancer therapy.

At normal oxygen levels, or normoxia, cells use the mitochondria to generate energy. When oxygen is not available, as under hypoxic conditions, cells shift to cytosolic glycolysis, an oxygen-independent pathway that converts glucose to pyruvate to produce ATP. The shift is accomplished by activating the transcription factor hypoxia-inducible factor-1 (HIF-1). HIF-1 is the master regulator of gene expression during hypoxia, and consists of a regulatory α subunit (HIFα) and a constitutive β subunit. Three forms of HIFα have been identified, of which HIF-1α and -2α contribute to cancer malignancy^1^,^2^,^3^,^4^. HIFα is suppressed in an oxygen-dependent manner by two hydroxylases, namely HIF prolyl hydroxylase and factor inhibiting HIF-1 (FIH-1). Prolyl hydroxylase promotes proteasomal degradation while FIH-1 inhibits transcriptional activity without affecting HIFα levels by preventing HIFα from binding to transcriptional co-factor p300/CBP^2^. Both enzymes are inactivated in hypoxic conditions to activate HIF-1.

Notably, HIF-1 is also activated during normoxia in some cells, including macrophages that require glycolysis to produce ATP, and cancer cells that exhibit the Warburg effect, a phenomenon in which glycolysis is enhanced even at normal oxygen levels^5^,^6^. In cancer cells, various oncogenic signalling pathways such as PI3K/AKT and Ras activate HIF-1 during normoxia by promoting the expression of HIF-1α, while inactivating mutations in the mitochondrial enzymes succinate dehydrogenase and fumarate hydratase stabilise HIF-1α^7^. In turn, HIF-1 contributes to the Warburg effect by promoting expression of glycolysis-related genes such as *SLC2A1*, *PGK1*, *PKM2*, *PDK1*, and *LDHA*^4^,^8^. However, the mechanisms that drive HIF-1 activation during normoxia are largely unclear, especially in terms of how HIF-1 is released from inhibition by prolyl hydroxylase or FIH-1.

Mint3 (also known as APBA3), a recently identified regulator of FIH-1, provides a clue^9^. Mint3 belongs to the X11 family of proteins, each of which contains a phosphotyrosine binding domain and two PDZ domains at the C-terminus^10^,^11^. Mint3 localises to the Golgi apparatus by interacting with membrane proteins such as amyloid precursor protein and furin^12^,^13^. The N-terminal region of Mint3 binds to and inhibits the enzymatic activity of FIH-1, resulting in HIF-1 activation even during normoxia without affecting HIF-1α levels^9^. Accordingly, Mint3 depletion in cancer cells reduces expression of HIF-1 target genes, glycolysis, and tumourigenicity^14^,^15^.

Remarkably, Mint3 activates HIF-1 only in particular types cancer cells and macrophages^14^,^16^,^17^, even though it is expressed almost ubiquitously^10^,^11^. These cells express membrane type-1 matrix metalloproteinase (MT1-MMP) and Mint3 activity is specifically regulated via MT1-MMP^14^,^15^,^18^. MT1-MMP is a well-characterised membrane protease that is up-regulated during the epithelial-mesenchymal transition in cancer cells^19^,^20^,^21^,^22^ to promote cell invasion^23^,^24^. MT1-MMP recruits cytoplasmic FIH-1 to the Golgi apparatus and sequesters it away from HIF-1 and into a complex with Mint3^18^,^25^. Thus, MT1-MMP is required for Mint3-mediated activation of HIF-1. In addition, mTOR complex 1, and pathways that activate it, enhance the activity of the MT1-MMP/Mint3 axis by phosphorylating Mint3^15^. In any case, the MT1-MMP/Mint3 axis activates HIF-1 only in normoxic conditions, and is superseded during hypoxia because of systemic inhibition of prolyl hydroxylase and FIH-1^14^.

In this study, we surveyed Mint3-binding proteins by yeast two-hybrid screening, and identified N-terminal EF-hand calcium binding protein 3 (NECAB3) as a regulator of the MT1-MMP/Mint3 axis. Importantly, inhibition of NECAB3 suppresses normoxic glycolysis in cancer cells and reduces tumourigenicity.

## Results

### NECAB3 binds Mint3

**Figure 1:**
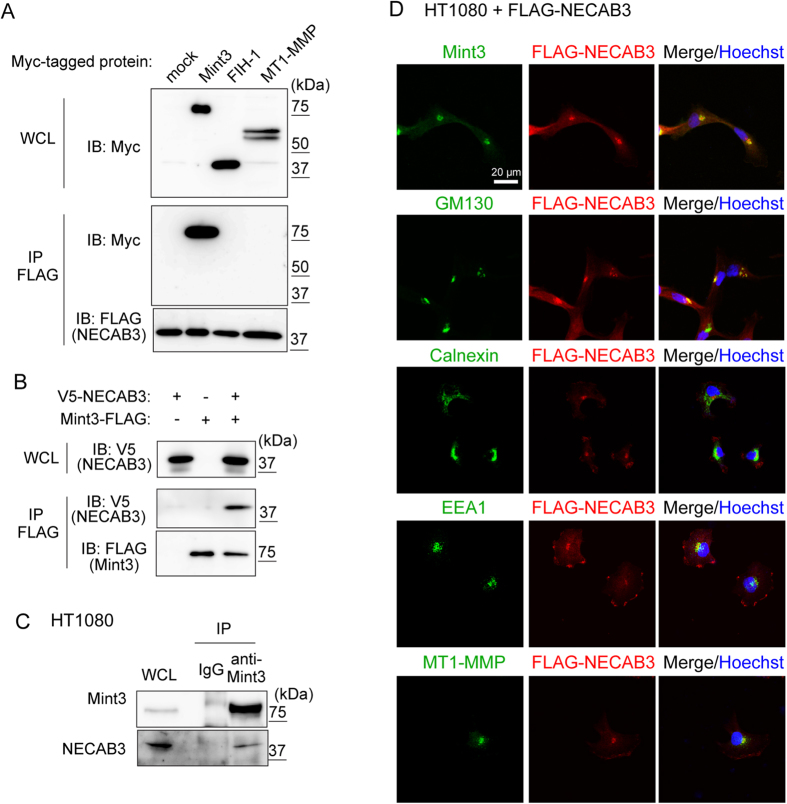
Identification of NECAB3 as a novel binding partner of Mint3. (**A**) NECAB3 binds to Mint3. FLAG-tagged NECAB3 was co-expressed with Myc-tagged Mint3, FIH-1, or MT1-MMP in 293FT cells. FLAG-tagged NECAB3 was immunoprecipitated (IP FLAG), and precipitates were analysed by immunoblotting (IB) using indicated antibodies. WCL, whole cell lysate. (**B**) FLAG-tagged Mint3 was co-expressed with V5-tagged NECAB3 in 293FT cells, and immunoprecipitated (IP FLAG). Precipitates were immunoblotted (IB) with indicated antibodies. WCL, whole cell lysate. (**C**) Whole cell lysates (WCL) from HT1080 cells were immunoprecipitated (IP) using control IgG or anti-Mint3, and immunoprecipitates were subjected to immunoblotting with indicated antibodies. (**D**) Immunostaining of FLAG-tagged NECAB3 (red) and endogenous Mint3, GM130, calnexin, EEA1, or MT1-MMP (green) in HT1080 cells. Nuclei were stained with Hoechst 33342 (blue).

Yeast two-hybrid screening using Mint3 as a bait identified eight potential Mint3-binding proteins, including NECAB3 (Supplementary Table S1). We characterised NECAB3 further, as it has been reported to bind APBA2, which also belongs to the Mint family, and to localise it like Mint3 to the Golgi apparatus^26^,^27^. To confirm interaction between Mint3 and NECAB3, FLAG-tagged NECAB3 was co-expressed with Myc-tagged Mint3, FIH-1, or MT1-MMP in mammalian 293FT cells. Mint3 was specifically co-precipitated with NECAB3 using beads coated with anti-FLAG (Fig. 1A). Conversely, FLAG-tagged Mint3 also co-precipitated V5-tagged NECAB3 (Fig. 1B). Interaction between endogenous Mint3 and NECAB3 was also confirmed by immunoprecipitation using human fibrosarcoma HT1080 lysates (Fig. 1C). Subsequently, FLAG-tagged NECAB3 was stably expressed in HT1080 cells, and was found to aggregate in the perinuclear region with Mint3 and GM130, a Golgi marker protein (Fig. 1D). Of note, we found that the staining pattern for Mint3 was quite distinct from those of the endoplasmic reticulum marker calnexin and the endosome marker EEA1, with which FLAG-tagged NECAB3 also partially co-localised (Fig. 1D). MT1-MMP, which co-localises with Mint3 at the Golgi apparatus^18^, also co-localised with FLAG-tagged NECAB3 (Fig. 1D). Taken together, the results indicate that NECAB3 binds and co-localises with Mint3 at the Golgi apparatus.

### NECAB3 depletion inhibits glycolysis in cancer cells

**Figure 2:**
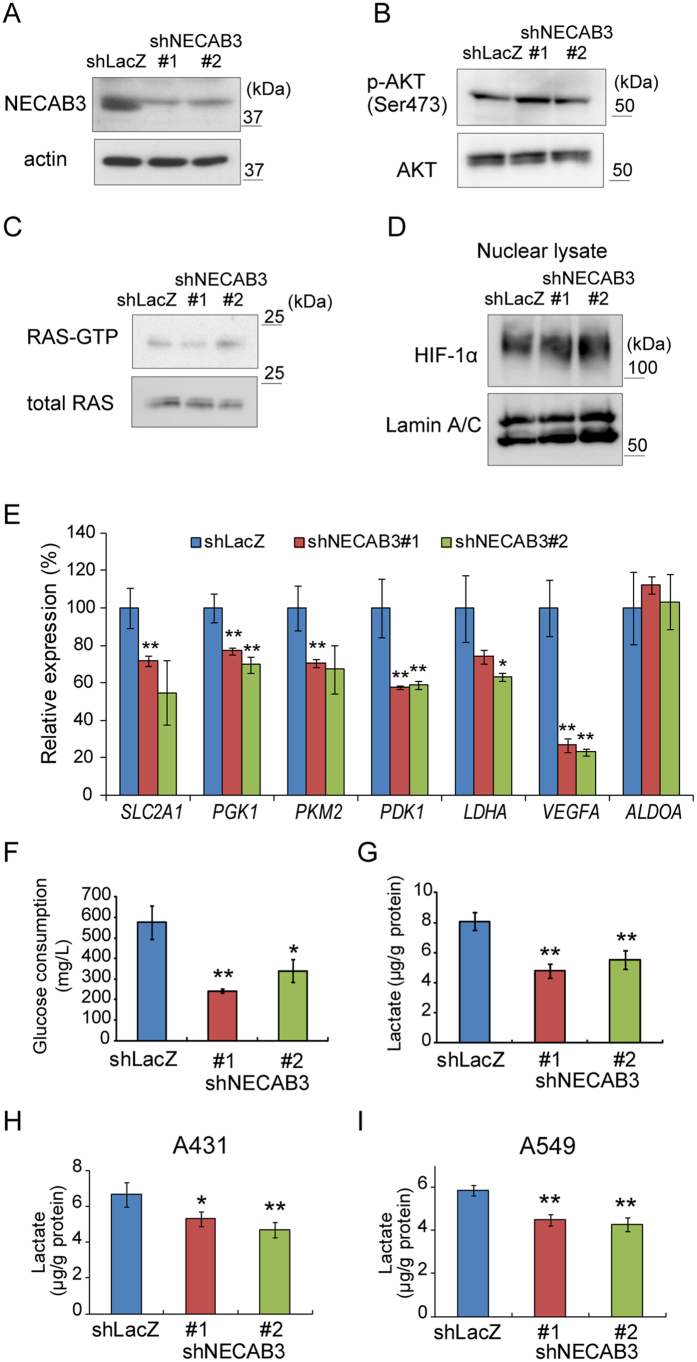
NECAB3 depletion attenuates glycolysis in HT1080 cells. (**A**) Immunoblotting of NECAB3 and actin in whole cell lysates from control (shLacZ) and NECAB3-depleted (shNECAB3) HT1080 cells. This experiment was repeated three times with consistent results. (**B**) Immunoblotting of unphosphorylated and Ser473-phosphorylated AKT in whole cell lysates from control (shLacZ) and NECAB3-depleted cells. Consistent results were obtained from three experiments. (**C**) Pull-down assay of active, GTP-bound Ras in whole cell lysates from control (shLacZ) and NECAB3-depleted cells. Results were consistent in three experiments. (**D**) Immunoblotting of HIF-1α in nuclear lysates from control and NECB3-depleted cells. Lamin A/C was used as loading control. Results from three experiments were comparable. (**E**) Expression of *SLC2A1*, *PGK1*, *PKM2*, *PDK1*, *LDHA*, *VEGFA*, and *ALDOA* in control and NECAB3-depleted cells was analysed by real-time PCR and normalised to *ACTB*. (**F**) Glucose consumption and (**G**) lactate production in control and NECAB3-depleted HT1080 cells. (**H,I**) Lactate production in control and NECAB3-depleted A431 (**H**) and A549 cells (**I**). In (**E–I**) error bars indicate s.d. (n = 3), and data were analysed by *t*-test. *p < 0.05 and **p < 0.01.

To investigate the role of NECAB3 in HIF-1 activation during normoxia, NECAB3 was depleted in HT1080 cells, in which HIF-1 is activated by the MT1-MMP/Mint3 axis even at normal oxygen levels^14^,^15^ (Fig. 2A). Similarly, oncogenic pathways such as PI3K/AKT and Ras promote expression of HIF-1α and thereby activate HIF-1 during normoxia^7^. However, NECAB3 depletion in HT1080 cells did not affect the phosphorylation state of AKT (Fig. 2B) and the abundance of active, GTP-bound Ras (Fig. 2C). Accordingly, NECAB3 depletion did not affect the abundance of HIF-1α (Fig. 2D), as observed when Mint3 is depleted^15^. However, expression of HIF-1 target genes, including *SLC2A1*, *PGK1*, *PKM2*, *PDK1*, *LDHA*, and *VEGFA,* were significantly diminished in NECAB3-depleted cells, as measured by real-time RT-PCR (Fig. 2E). In contrast, NECAB3 depletion did not affect expression of *ALDOA,* which encodes a glycolysis enzyme but is not a HIF-1 target gene (Fig. 2E). Thus, NECAB3 depletion specifically suppressed expression of HIF-1 target genes.

HIF-1 activation by Mint3 promotes glycolysis in cancer cells, even at normal oxygen levels^14^. Thus, glucose consumption and lactate production due to glycolysis were analysed in HT1080 cells from which NECAB3 had been knocked down. As expected, both glucose consumption and lactate production decreased significantly in NECAB3-depleted cells (Fig. 2F,G). Similar results were obtained in human squamous cell carcinoma A431 cells and human lung adenocarcinoma A549 cells (Fig. 2H,I). These results indicate that NECAB3 promotes glycolysis during normoxia in various cancer cells that exhibit the Warburg effect.

### Suppression of glycolysis by NECAB3 depletion requires the MT1-MMP/Mint3 axis

**Figure 3:**
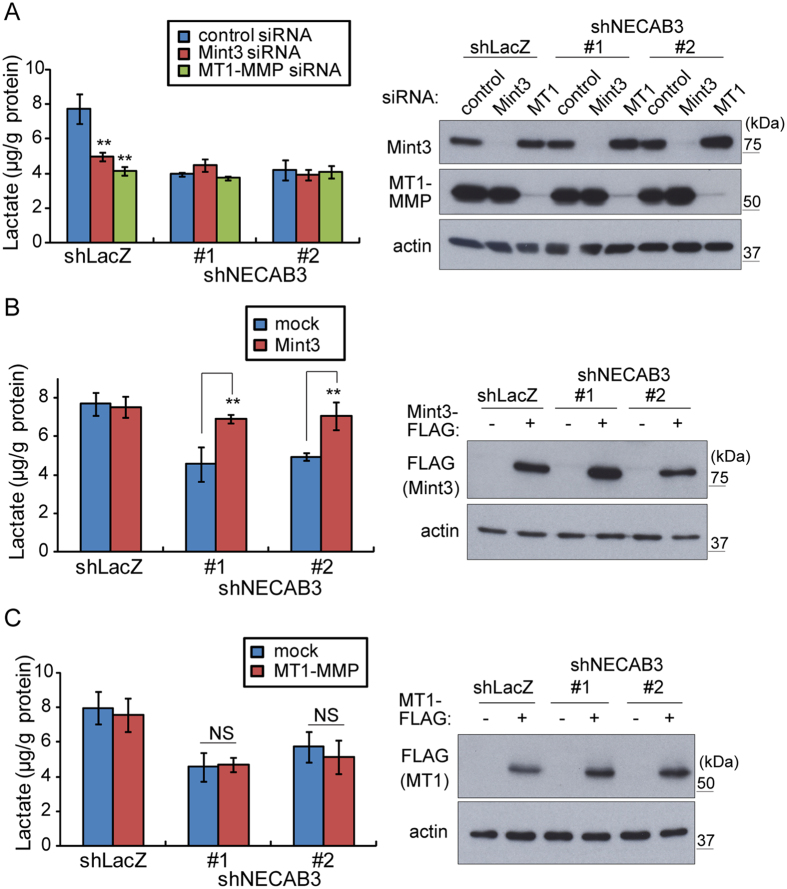
NECAB3 promotes glycolysis via the MT1-MMP/Mint3 axis in HT1080 cells. (**A**) Lactate production, left, in HT1080 cells treated with control or NECAB3 shRNA in combination with control, Mint3, or MT1-MMP siRNAs. siRNA knockdown was analysed by immunoblotting, right. (**B,C**) Lactate production, left, in control and NECAB3-depleted HT1080 cells transfected with mock or FLAG-tagged Mint3 expression vector (**B**) or with mock or FLAG-tagged MT1-MMP expression vector (**C**). Expression of FLAG-tagged Mint3 (**B**) and FLAG-tagged MT1-MMP was analysed by immunoblotting **(C)**, right. Error bars in (**A–C**) are s.d. (n = 3), and data were analysed by *t*-test. **p < 0.01 and NS, not significant.

Mint3 requires MT1-MMP to activate HIF-1 in cancer cells and enhance glycolysis^14^,^15^. Therefore, lactate production in control or NECAB3-depleted HT1080 cells was evaluated following transient siRNA knockdown of Mint3 or MT1-MMP. Knockdown of Mint3 or MT1-MMP decreased lactate production in control cells, as previously reported^14^,^15^ (Fig. 3A, shLacZ), without affecting AKT phosphorylation state and Ras activity (Supplementary Fig. S1A). In contrast, lactate production in NECAB3-depleted HT1080 cells was not affected by additional knockdown of Mint3 or MT1-MMP (Fig. 3A, shNECAB3 #1 and #2). We further examined whether overexpression of Mint3 or MT1-MMP restores lactate production in NECAB3-depleted HT1080 cells. As we reported previously, inhibition of FIH-1 by endogenous Mint3 requires MT1-MMP, although overexpression of Mint3 is sufficient by itself to inhibit FIH-1 because of altered cytoplasmic localisation^9^,^18^. Accordingly, overexpression of Mint3 (Fig. 3B), but not MT1-MMP (Fig. 3C), restored lactate production in NECAB3-depleted HT1080 cells to basal levels (Fig. 3B), also without perturbing AKT phosphorylation state and Ras activity (Supplementary Fig. S1B). Taken together, the data suggest that glycolysis deficiency due NECAB3 knockdown depends on the MT1-MMP/Mint3 axis, but is not rescued by MT1-MMP overexpression.

### NECAB3 depletion destabilises the complex between Mint3 and FIH-1

**Figure 4:**
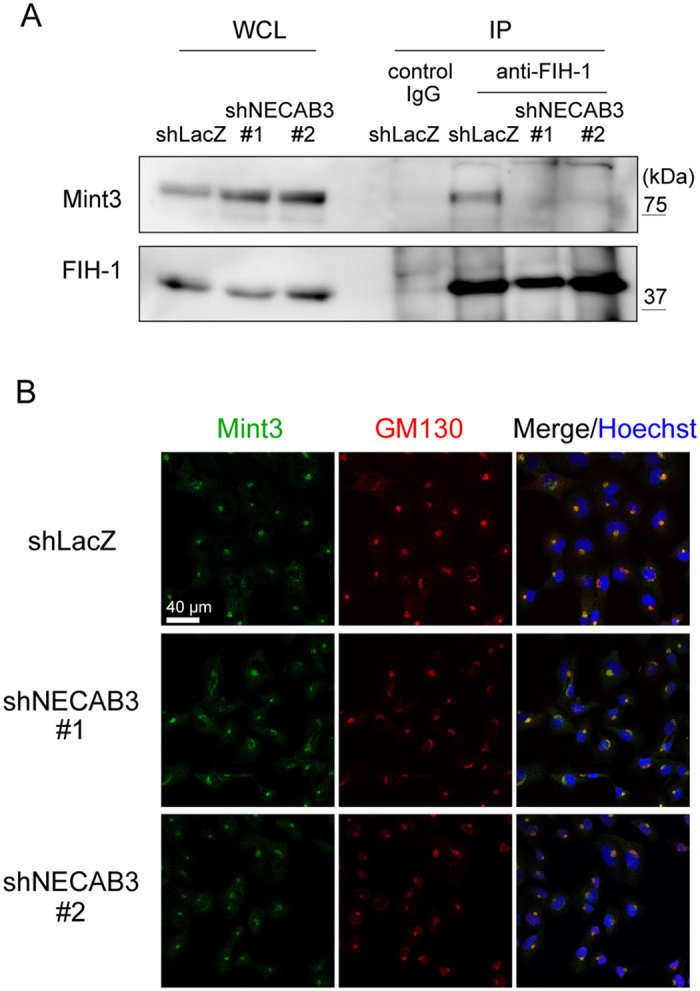
NECAB3 depletion destabilises the complex between Mint3 and FIH-1. (**A**) Immunoprecipitates (IP) of FIH-1 from whole cell lysates (WCL) were analysed by immunoblotting (IB) for FIH-1 and Mint3. (**B**) Immunostaining of Mint3 (green) and GM130 (red) in control and NECAB3-depleted HT1080 cells. Nuclei were stained with Hoechst 33342 (blue).

Mint3 binds and inhibits FIH-1 to activate HIF-1 during normoxia^9^. Because NECAB3 depletion reduced expression of HIF-1 target genes (Fig. 2E), we examined whether NECAB3 depletion affects the interaction between Mint3 and FIH-1. Mint3 was co-precipitated with FIH-1 in control cells (Fig. 4A, shLacZ), but not in NECAB3-depleted HT1080 cells (Fig. 4A, shNECAB3 #1 and #2), suggesting that NECAB3 stabilised the interaction between Mint3 and FIH-1. We then investigated the possibility that NECAB3 depletion redistributes Mint3 away from the Golgi apparatus. Confocal microscopy indicated that NECAB3 depletion did not affect Mint3 localisation in HT1080 cells (Fig. 4B and Supplementary Fig. S2). Thus, NECAB3 depletion interferes with Mint3-FIH-1 interaction without displacing Mint3 from the Golgi apparatus.

### NECAB3 forms a ternary complex with Mint3 and FIH-1

**Figure 5:**
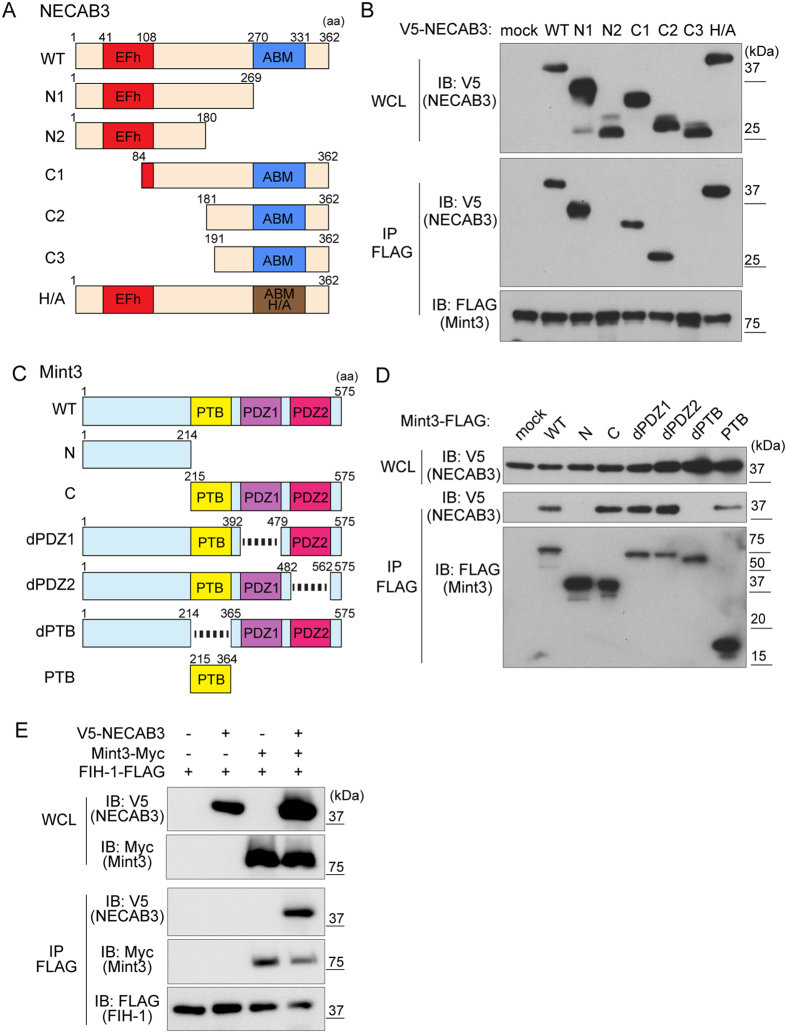
NECAB3 interacts with FIH-1 indirectly via Mint3. (**A**) Domain structure of NECAB3 and mutants constructed. NECAB3 contains an EF-hand (EFh) and an antibiotic biosynthesis monooxygenase (ABM) domain. In the H/A mutant, the catalytic His^324^ residue is replaced with Ala. Amino acid (aa) positions are indicated. (**B**) Immunoprecipitates (IP) of FLAG-tagged Mint3 were analysed by immunoblotting (IB) for V5-tagged NECAB3 mutants. (**C**) Domain structure of Mint3 and mutants constructed. Mint3 contains a phosphotyrosine-binding (PTB) and two PDZ domains. Amino acid (aa) positions are indicated. (**D**) Immunoprecipitates (IP) of FLAG-tagged Mint3 mutants were analysed by immunoblotting (IB) for V5-tagged NECAB3. WCL, whole cell lysate. (**E**) Immunoprecipitates (IP) of FLAG-tagged FIH-1 were analysed by immunoblotting (IB) for V5-tagged NECAB3 and Myc-tagged Mint3. WCL, whole cell lysate.

To map Mint3-binding sites, truncation mutants of NECAB3 were expressed in 293FT cells. NECAB3 contains an EF-hand domain at the N-terminus and an antibiotic biosynthesis monooxygenase domain at the C-terminus (Fig. 5A). Immunoprecipitation assays revealed that a fragment consisting of amino acids 181–190 is critical for binding Mint3, but not the EF-hand or the monooxygenase domains (Fig. 5B, C2 and C3). The homologous monooxygenase domain in *Streptomyces* synthesises several antibiotics via a conserved, catalytic histidine^28^. However, a mutated form of NECAB3, in which the catalytic His^324^ in the monooxygenase domain is replaced with Ala, bound Mint3 (Fig. 5B, H/A). Hence, the data indicate that Mint3 binding depends neither on sequences in the monooxygenase domain nor on enzymatic activity.

Conversely, FLAG-tagged truncation mutants of Mint3 (Fig. 5C) were expressed in 293FT cells and subjected to immunoprecipitation with V5-tagged NECAB3. The N-terminus of Mint3, which binds to FIH-1^9^, did not bind to NECAB3, in contrast to the C-terminus (Fig. 5D, N and C). As noted, the C-terminus of Mint3 consists of a phosphotyrosine-binding domain and two PDZ domains (Fig. 5C). These domains were individually deleted, and only deletion of the phosphotyrosine-binding domain abolished binding (Fig. 5D, dPTB). On the other hand, the phosphotyrosine-binding domain by itself bound to NECAB3 (Fig. 5D, PTB), suggesting that it is necessary and sufficient for binding.

Unlike NECAB3, FIH-1 binds to the N-terminus of Mint3^9^, and we examined whether NECAB3 binds to FIH-1 indirectly via Mint3. Indeed, NECAB3 was immunoprecipitated with FIH-1 only when Mint3 was expressed in 293FT cells (Fig. 5E), suggesting that NECAB3 formed a ternary complex with Mint3 and FIH-1.

### NECAB3 mutants that bind Mint3 but lack an intact monooxygenase domain are dominant negative

**Figure 6:**
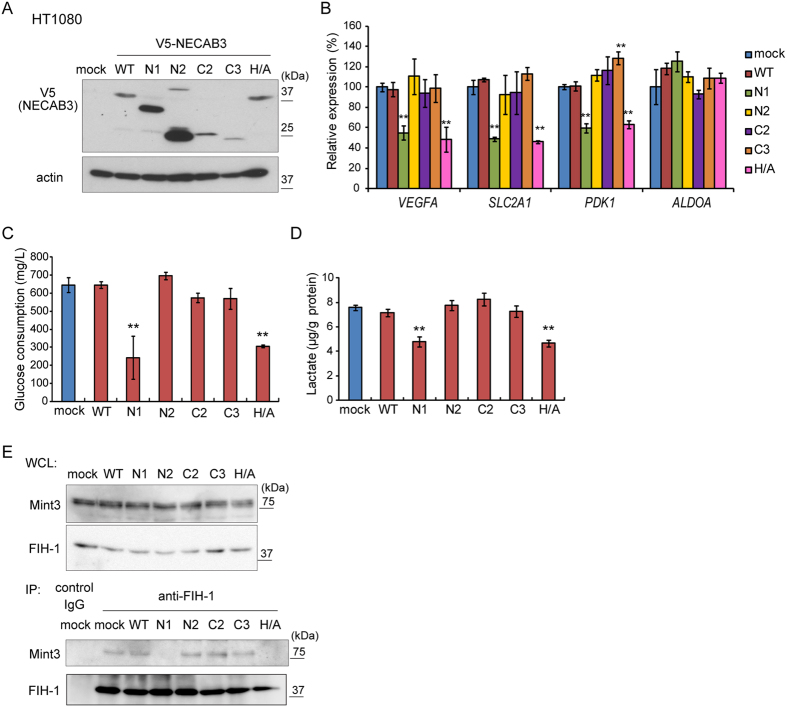
NECAB3 mutants without an intact monooxygenase domain are dominant negative against endogenous NECAB3. (**A**) Expression of V5-tagged NECAB3 and mutants in HT1080 cells. (**B**) Expression of *VEGFA*, *SLC2A1*, *PDK1*, and *ALDOA* was analysed by real-time PCR in HT1080 cells expressing exogenous NECAB3 or its mutants. Expression was normalised to *ACTB*. Error bars indicate s.d. (n = 3), and data were analysed by *t*-test. *p < 0.05 and **p < 0.01. (**C**,**D**) Glucose consumption **(C)** and lactate production (**D**) in HT1080 cells expressing exogenous NECAB3 or its mutants. Error bars represent s.d. (n = 3), and data were compared pairwise by *t*-test. **p < 0.01. (**E**) Immunoprecipitates (IP) of FIH-1 from whole cell lysates (WCL) were analysed by immunoblotting (IB) for FIH-1 and Mint3.

As NECAB3 depletion suppressed HIF-1 activity and glycolysis during normoxia in HT1080 cells (Fig. 2E–G), we tested whether exogenous expression of NECAB3 or its mutants boosts HIF-1 activity. V5-tagged wild type or mutant NECAB3 was stably expressed in parental HT1080 cells (Fig. 6A), and real-time RT-PCR indicated that exogenous wild type NECAB3 did not stimulate expression of HIF-1 target genes (Fig. 6B, WT). Remarkably, NECAB3 mutants that bind Mint3 but lack an intact monooxygenase domain specifically suppressed expression of HIF-1 target genes (Fig. 6B, N1 and H/A). Accordingly, glucose consumption and lactate production diminished (Fig. 6C,D). In addition, these mutants did not further reduce glycolysis activity in NECAB3-depleted HT1080 cells (Supplementary Fig. S3). Thus, we hypothesised that these mutants are dominant negative against endogenous NECAB3. Indeed, immunoprecipitation experiments demonstrated that these mutants destabilised the interaction between Mint3 and FIH-1 (Fig. 6E) to a similar extent as NECAB3 depletion (Fig. 4A), and in a manner that also depended on Mint3 (Supplementary Fig. S4). These results suggest that NECAB3 mutants that bind Mint3 but lack an intact monooxygenase domain inhibit endogenous NECAB3.

### NECAB3 depletion reduces tumourigenicity

**Figure 7:**
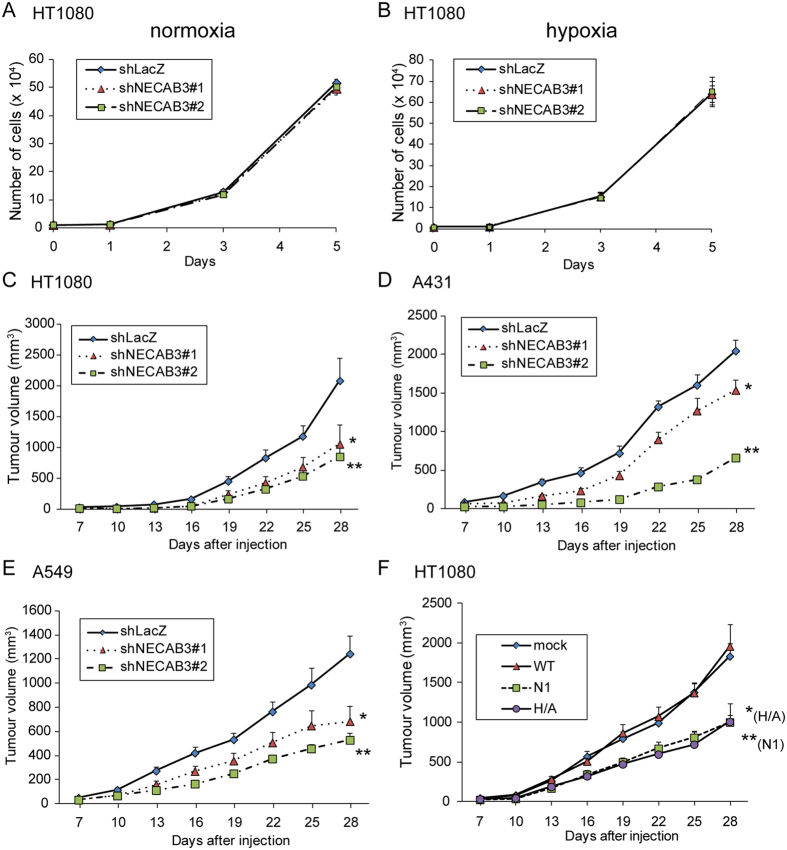
NECAB3 inhibition attenuates growth of cancer cells implanted *in vivo*. (**A**,**B**) Growth of control and NECAB3-depleted HT1080 cells in culture dishes kept at normoxic (**A**) or hypoxic (**B**) conditions. Error bars indicate s.d. (n = 3). (**C**) Growth of NECAB3-depleted HT1080 cells subcutaneously implanted into immunodeficient mice. Error bars indicate s.e.m. (n = 11), and data were analysed by Mann-Whitney *U*-test. *p < 0.05 and **p < 0.01. (**D**,**E**) Growth of NECAB3-depleted A431 (**D**) and A549 cells (**E**) implanted into immunodeficient mice. Error bars indicate s.e.m. (n = 6), and data were analysed by Mann-Whitney *U*-test. *p < 0.05 and **p < 0.01. (**F**) Growth of HT1080 cells expressing exogenous NECAB3 and its mutants implanted into immunodeficient mice. Error bars indicate s.e.m. (n = 12), and data were analysed by Mann-Whitney *U*-test. *p < 0.05 and **p < 0.01.

Next, we examined whether NECAB3 depletion reduces tumourigenicity. We first verified that NECAB3 depletion did not affect cell growth *in vitro* under normoxic and hypoxic conditions (Fig. 7A,B), as observed in Mint3 depletion^14^,^15^. Control (shLacZ) and NECAB3-depleted (shNECAB3) HT1080 cells were then grafted into immunodeficient mice. NECAB3 depletion decreased subcutaneous tumour growth to approximately 40–50% of control cells (Fig. 7C). Similarly, NECAB3 depletion decreased tumour growth from A431 and A549 cells (Fig. 7D,E) to a comparable extent as Mint3 depletion (Supplementary Fig. S5). Exogenous expression of NECAB3 dominant negative mutants also decreased tumour growth to approximately 50% (Fig. 7F, N1 and H/A). Thus, NECAB3 depletion by expression of shRNAs or dominant negative mutants reduced tumourigenicity.

### Expression of NECAB3, Mint3, and MT1-MMP in human tumour and normal tissues

**Figure 8:**
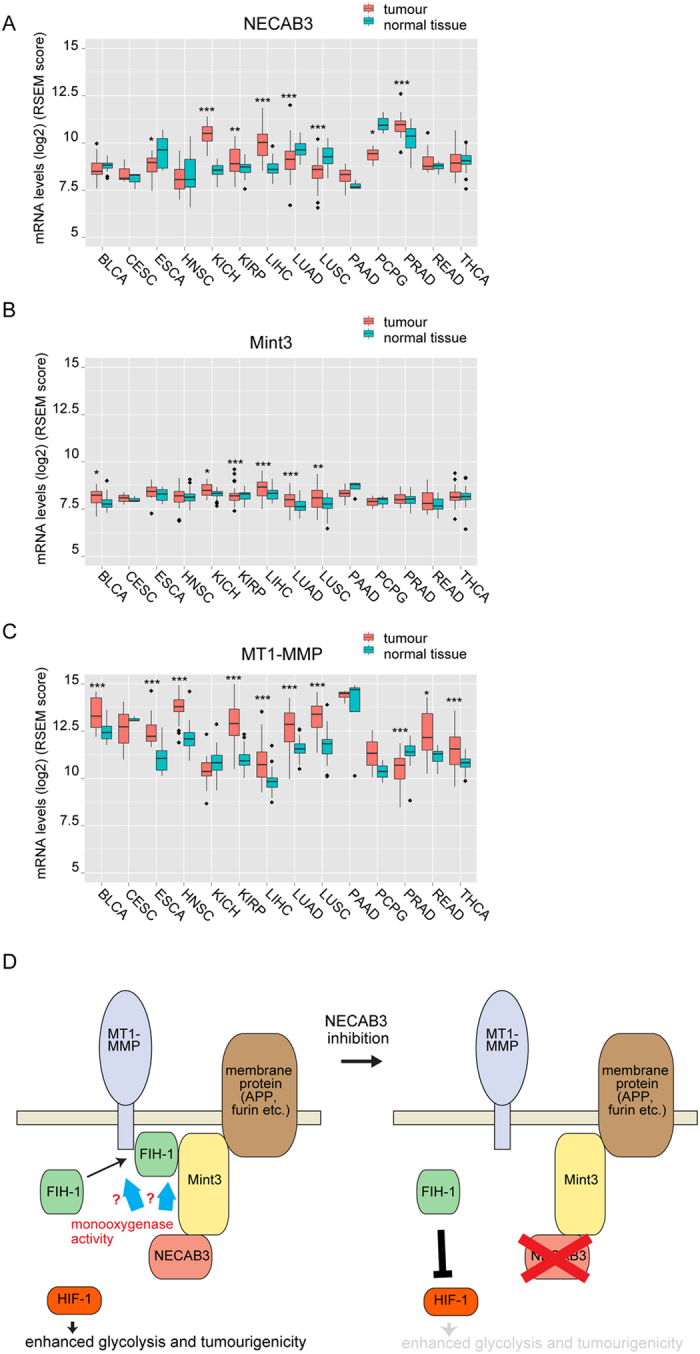
Expression of NECAB3, Mint3, and MT1-MMP in human tumour and normal tissues. **(A–C**) Expression of NECAB3 (**A**), Mint3 (**B**), and MT1-MMP (**C**) in TCGA datasets. BLCA, urothelial bladder cancer; CESC, cervical cancer; ESCA, oesophageal cancer; HNSC, head and neck squamous cell carcinoma; KICH, chromophobe renal cell carcinoma; KIRP, papillary kidney carcinoma; LIHC, liver hepatocellular carcinoma; LUAD, lung adenocarcinoma; LUSC, lung squamous cell carcinoma; PAAD, pancreatic ductal adenocarcinoma; PCPG, phaeochromocytoma and paraganglioma; PRAD, prostate adenocarcinoma; READ, rectal adenocarcinoma; THCA, papillary thyroid carcinoma. Data were analysed by *t*-test. *p < 0.05; **p < 0.01; ***p < 0.001. (**D**) Model of NECAB3-mediated glycolysis and tumourigenicity. NECAB3 binds Mint3 and, if enzymatically active, stabilises the complex between Mint3 and FIH-1. When NECAB3 is depleted or a dominant negative mutant is expressed, the interaction between Mint3 and FIH-1 is abolished. As a result, FIH-1 suppresses HIF-1 activity, and decreases glycolysis and tumourigenicity.

Finally, we analysed expression of NECAB3, Mint3, and MT1-MMP in human tumour and normal tissues using mRNA-sequencing datasets from The Cancer Genome Atlas (TCGA). NECAB3 expression was significantly increased in five tumours (urothelial bladder cancer, chromophobe renal cell carcinoma, papillary kidney carcinoma, liver hepatocellular carcinoma, and prostate adenocarcinoma), but significantly decreased in four (oesophageal cancer, lung adenocarcinoma, lung squamous cell carcinoma, and phaeochromocytoma and paraganglioma) (Fig. 8A). Mint3 was modestly induced in some tumours (Fig. 8B), while MT1-MMP was elevated in many (Fig. 8C). Interestingly, NECAB3, Mint3, and MT1-MMP were induced in three tumours (urothelial bladder cancer, papillary kidney carcinoma, liver hepatocellular carcinoma), implying that NECAB3-mediated HIF-1 activation might play some role in these cancers.

## Discussion

Cancer cells activate HIF-1 to promote glycolysis during normoxia through mechanisms that are not fully understood. Our results suggest that NECAB3, a novel Mint3-binding protein, activates HIF-1 to promote normoxic glycolysis and tumourigenicity. NECAB3 did not directly bind to FIH-1, an inhibitor of HIF-1, but formed a ternary complex with Mint3 and FIH-1. NECAB3 depletion prevented formation of the complex between Mint3 and FIH-1, suppressed expression of HIF-1 target genes and glycolysis during normoxia, and reduced tumourigenicity. Though exogenous expression of NECAB3 did not further enhance HIF-1 activity, NECAB3 mutants that binds Mint3 but lacks an intact monooxygenase domain had a dominant negative effect on endogenous NECAB3 (Fig. 6A–E). These results are summarised in Fig. 8D.

Notably, an intact monooxygenase domain is necessary to promote glycolysis during normoxia (Supplementary Fig. S3A–C). Thus, NECAB3 monooxygenase activity is also a possible target to control MT1-MMP/Mint3-induced activation of HIF-1 in cancer cells. We anticipate that further studies will identify the direct target(s) of oxygenation by NECAB3. On the other hand, we previously demonstrated that mTOR complex 1 phosphorylates Mint3 and thereby stabilises the interaction between Mint3 and FIH-1^15^. However, NECAB3 depletion did not affect the phosphorylation state of Mint3 (Supplementary Fig. S6), indicating that NECAB3 supports the interaction between Mint3 and FIH-1 independently of mTOR complex 1.

There are three known NECAB proteins. NECAB1 and 2 are expressed predominantly in the brain, whereas NECAB3 is expressed not only in the brain but also in the heart, muscle, and pancreas^26^,^29^. These proteins are thought to be involved in amyloid precursor protein metabolism^26^,^30^, but their role in cancer cells has not been investigated. Using RT-PCR, we found NECAB2 and NECAB3, but not NECAB1, to be expressed in cancer cells (Supplementary Fig. S7). However, knockdown of NECAB3 alone was sufficient to inhibit normoxic glycolysis and reduce tumourigenicity. Thus, NECAB3 seems to function distinctly from other NECABs, at least in cancer cells.

NECAB3 was previously identified as a binding partner of APBA2 (also known as X11L)^26^. Like Mint3, APBA2 is a member of the X11 protein family, and contains one PTB domain and two PDZ domains at the C-terminus. However, APBA2 binds NECAB3 via the N-terminal region (1–367 AA)^26^ while Mint3 binds to NECAB3 via the phosphotyrosine-binding domain (Fig. 5D). In addition, APBA2 is predominantly expressed in the brain, while Mint3 is ubiquitously expressed^10^. Thus, it is unclear whether APBA2 controls NECAB3 function, but such regulation might be restricted to the brain, if at all.

HIF-1 is precisely and specifically regulated in cells, because it controls various cellular activities, including the expression of a wide array of genes. Thus, HIF-1α is regulated by various post-transcriptional modifications such as hydroxylation, acetylation, and sumoylation^7^,^31^, and cancer cells seem to co-opt these mechanisms to promote cell survival and proliferation. Mint3-dependent activation of HIF-1 is additionally regulated, so far as known, by MT1-MMP, by the AKT/mTOR signalling pathway, and, now, by NECAB3^14^,^15^. However, we identified seven other potential Mint3-binding proteins by yeast two-hybrid screens (Supplementary Table S1), and further characterisation of these candidates may illuminate the precise regulatory mechanism that drives HIF-1 activation at normal oxygen levels.

In summary, we demonstrated that NECAB3, a novel Mint3-binding protein, plays an essential role in normoxic HIF-1 activation by MT1-MMP/Mint3. Hence, further studies on the interaction between the MT1-MMP/Mint3 axis and NECAB3 may uncover the molecular basis of the Warburg effect. Finally, targeting NECAB3, especially its monooxygenase activity, may prove useful as a cancer therapeutic strategy.

## Methods

### Yeast two-hybrid screens

Yeast two-hybrid screens against a randomly primed cDNA library from human placenta were performed by Hybrigenics (Paris, France), using Mint3 as a bait. Protein-protein interactions were individually assigned a statistical confidence score as described previously^32^.

### Cell culture

HT1080 human fibrosarcoma, A431 human epidermoid carcinoma, and A549 human lung carcinoma cells were purchased from the American Type Culture Collection (Manassas, VA, USA). 293FT cells, which are derived from HEK293 human embryonic kidney cells and express the simian virus large T antigen, were purchased from Life Technologies (Carlsbad, CA). Cells were cultured at 37 °C and humidified 5% CO_2_ in DMEM (HT1080, A431, and A549) or high-glucose DMEM (293FT) containing 10% foetal bovine serum, 100 units/mL penicillin, and 100 μg/mL streptomycin (Sigma-Aldrich, St Louis, MO, USA). For experiments in hypoxic conditions, cells were cultured with 1% O_2_ and 5% CO_2_ in a model 9200 incubator (Wakenyaku, Tokyo, Japan).

### Vector construction

Control shRNA against LacZ had sequence 5′-gcuacacaaaucagcgauuucgaaaaaucgcugauuuguguag-3′. NECAB3 was silenced with 5′-gaagaaagcaggacccucgcuacgaauagcgaggguccugcuuuc-3′ (#1) and 5′-ggguugguuugcaugucauuucgaaaaaugacaugcaaaccaaccc-3′ (#2). Targeted gene sequences were subcloned as deoxyribose fragments into pENTR/U6 TOPO (Life Technologies) and recombined into the lentivirus vector pLenti6 BLOCKiT. Human *NECAB3* cDNA was obtained from HT1080 cells by reverse transcription PCR, and constructs expressing mutant NECAB3 or Mint3 were generated using PCR-based methods. These fragments were subcloned into pENTR/D-TOPO (Life Technologies) and recombined into the lentivirus vector pLenti6 or the mammalian expression vector pcDNA3.2, as described previously^9^,^14^,^15^. Expression vectors for MT1-MMP, Mint3, and FIH-1 were constructed as previously described^9^. Lentiviral vectors were generated and used according to the manufacturer’s instructions.

### siRNA knockdown

Knockdown by siRNA was carried out using Lipofectamine™ RNAiMAX (Life Technologies) as previously described^14^. Mint3 was silenced with a mixture of 5′-gauggaacuugaugaguca-3′, 5′-gggaggugcaccucgagaa-3′, and 5′-gguucuugguccuguauga-3′, while MT1-MMP was knocked down with a mix of 5′-ggauggacacggagaauuu-3′, 5′-gcgaugaagucuucacuua-3′, and 5′-ggguagagacccugagaca-3′. Control siRNA consisted of a mixture of 5′-auccgcgcgauaguacgua-3′, 5′-uuacgcguagcguaauacg-3′, and 5′-uauucgcgcguauagcggu-3′.

### Cell proliferation

Cells (1 × 10^4^) were seeded in a plastic dish, cultured at 37 °C in a humidified CO_2_ incubator, and counted periodically using a Coulter counter (Beckman, Fullerton, CA).

### Immunoblotting

Cells were lysed with lysis buffer (150 mM NaCl, 50 mM Tris pH 8.0, and 1% NP-40), and centrifuged at 20,000 × *g* for 15 min at 4 °C. Supernatants were collected and total protein content was measured using Bradford assay (Bio-Rad, Hercules, CA). Nuclear lysates were prepared using a Nuclear Extract Kit (Active Motif, Carlsbad, CA). Extracts were separated by SDS-PAGE, transferred to membranes, and analysed by immunoblotting using antibodies against V5 (Life Technologies), Myc (Medical & Biological Laboratories, Nagoya, Japan), FLAG epitope M2 (Sigma-Aldrich), MT1-MMP (Merck Millipore, Billerica, MA), Mint3 (BD Biosciences, Franklin Lakes, NJ), FIH-1 (Santa Cruz Biotechnology, Dallas, TX), actin (Merck Millipore), HIF-1α (BD Biosciences), lamin A/C (Cell Signaling Technology, Danvers, MA), AKT (Cell Signaling Technology), phospho-AKT (Ser473) (Cell Signaling Technology), and NECAB3. Anti-NECAB3 was generated in chicken by SCRUM Inc. (Tokyo, Japan) using His_6_-NECAB3 as immunogen, and the IgY fraction was purified from immunised egg lysates. Pan-Ras Activation Assay Kit (Cell Biolabs, San Diego, CA) was used to detect active Ras according to the manufacturer’s instructions.

### Immunoprecipitation

Immunoprecipitation was performed as previously described^9^,^15^. Briefly, 293FT cells were co-transfected using Lipofectamine™ 2000 (Life Technologies) with expression plasmids encoding a V5-tagged construct, a FLAG-tagged construct, and a Myc-tagged construct. Cells were lysed in lysis buffer 24 h after transfection, and centrifuged at 20,000 × *g* for 15 min at 4 °C. Supernatants were collected and incubated with beads conjugated to anti-FLAG M2 (Sigma-Aldrich). Beads were washed, and bound proteins were eluted with FLAG peptide, and analysed by immunoblotting. To detect interaction between FIH-1 and Mint3, HT1080 cells were lysed with lysis buffer, and centrifuged at 20,000 × *g* for 15 min at 4 °C. Supernatants were collected and incubated overnight at 4 °C with rabbit anti-FIH-1 polyclonal antibody (Novus Biologicals, Littleton, CO, USA) or control rabbit IgG (Sigma-Aldrich). Lysates were then incubated with protein G-Sepharose (GE Healthcare, Little Chalfont, UK) for 30 min at 4 °C. Beads were washed four times with lysis buffer, and proteins were eluted with Laemmli sample buffer, and analysed by immunoblotting.

### Lactic acid production and glucose consumption

Lactic acid levels and glucose consumption were measured using the L-Lactic Acid Kit (R-Biopharm, Darmstadt, Germany) and Glucose (GO) Assay Kit (Sigma-Aldrich), as previously described^15^.

### Immunostaining

Cells were fixed with 4% paraformaldehyde and permeabilised with 0.01% Triton X-100 for 10 min. After blocking for 30 min in phosphate-buffered saline containing 5% goat serum and 3% bovine serum albumin, cells were incubated for 1 h with a mixture of rabbit antibodies against FLAG (polyclonal, Sigma-Aldrich) or GM130 (BD Biosciences), and mouse antibodies against Mint3 (BD Biosciences), GM130 (BD Biosciences), calnexin (Abcam, Cambridge, U.K.), EEA1 (BD Biosciences), or MT1-MMP (Merck Millipore). After washing three times with phosphate-buffered saline, cells were incubated for 1 h with anti-mouse IgG and anti-rabbit antibody conjugated to Alexa Fluor 488 and 594 (Life Technologies). Cells were washed five times with phosphate-buffered saline, mounted on slides, and imaged by confocal microscopy (Nikon A1, Nikon, Tokyo, Japan).

### RNA isolation, reverse transcription (RT), and real-time PCR

Total RNA was isolated using TRIzol reagent (Life Technologies), and reverse transcribed as described^33^ using SuperScript II (Life Technologies) and random primers. Products were then analysed by real-time PCR on an ABI3700 system (ABI, Foster City, CA) using SYBR green I Mix (ABI) and primers specific for *ACTB* (sense, 5′-gggacgacatggagaaaatc-3′ and antisense, 5′-gggtgttgaaggtctcaaac-3′), *SLC2A1* (sense, 5′-gggcatgtgcttccagtatgt-3′ and antisense, 5′-accaggagcacagtgaagat-3′), *PGK1* (sense, 5′-gcatacctgctggctggatg-3′ and antisense, 5′-cccacaggaccattccacac-3′), *PKM2* (sense, 5′-gcctgtcatctgtgctactc-3′ and antisense, 5′-ctccagacagcatgatgcag-3′), *PDK1* (sense, 5′-tcctgtcaccagccagaatg-3′ and antisense, 5′-cttcctttgccttttccacc-3′), *LDHA* (sense, 5′-ctccaagctggtcattatcacg-3′ and antisense, 5′-agttcgggctgtattttacaaca-3′), *ALDOA* (sense, 5′-ggctgcagatgagtccactg-3′ and antisense, 5′-tcgtcagctgtcagcagcag-3′), and *VEGFA* (sense, 5′-ctccaccatgccaagtggtc-3′ and antisense, 5′-actcctggaagatgtccacc-3′). PCR products were sequenced, and homogeneity was confirmed by the dissociation temperature, as measured by SYBR green I fluorescence.

### Tumourigenicity

Experimental protocols were approved by the the Animal Care and Use Committees of The Institute of Medical Science, University of Tokyo (permit number: PA13-115), and all experiments were conducted according to the institutional ethical guidelines for animal experiments and the safety guidelines for gene manipulation experiments by The Institute of Medical Science, University of Tokyo. Briefly, 1 × 10^6^ (HT1080 and A431) or 5 × 10^5^ (A549) cells were injected subcutaneously into 6-week-old female BALB/c nude mice (CLEA Japan, Tokyo, Japan). Dimensions of the resulting tumours were measured with a calliper, and volumes were calculated according to V = (L × W^2^)/2, where V is the volume (mm^3^), L is the largest diameter (mm), and W is the smallest diameter (mm).

### Gene expression in human tumour and normal tissues

Expression of NECAB3, Mint3, and MT1-MMP in human tumour and normal tissues was compared using TCGA GDAC Firehose standard data version “2015_11_01 stddata Run”. Data were downloaded from the Broad TCGA GDAC web site (http://gdac.broadinstitute.org/), and cancer types with less than three samples were excluded from analysis. mRNA levels were estimated by RSEM^34^.

### Statistical analysis

Groups were compared pairwise using two-sided *t*-test or Mann-Whitney *U*-test.

## Additional Information

**How to cite this article**: Nakaoka, H. J. *et al.* NECAB3 Promotes Activation of Hypoxia-inducible factor-1 during Normoxia and Enhances Tumourigenicity of Cancer Cells. *Sci. Rep.* **6**, 22784; doi: 10.1038/srep22784 (2016).

## Supplementary Material

Supplementary Information
