# Supplementary material for: NECAB3 Promotes Activation of Hypoxia-inducible factor-1 during Normoxia and Enhances Tumourigenicity of Cancer Cells
Source: Sci Rep. 2016 Mar 7;6:22784. doi: 10.1038/srep22784 (PMC4780031; doi:10.1038/srep22784)
Supplement: Supplementary Information [file srep22784-s1.pdf]

NECAB3 Promotes Activation of Hypoxia-inducible factor-1 during Normoxia and Enhances Tumourigenicity of Cancer Cells

Hiroki J. Nakaoka[1,2], Toshiro Hara[1], Seiko Yoshino[1,2], Akane Kanamori[2], Yusuke Matsui[3], Teppei Shimamura[3], Hiroshi Sato[4], Yoshinori Murakami[2], Motoharu Seiki[1,5] & Takeharu Sakamoto[1,2]

Supplementary Materials and Methods

shRNA sequences. shRNAs against Mint3 had sequence 5'-gaugauggcgguggugacgggacgaaucccgucaccaccgccauca-3' (#1) and 5'-gcgguucuugguccuguaugacgaaucauacaggaccaagaaccgc-3' (#2).

Primer sequences. Primers specific for *NECAB1* (sense, 5'-ttcctcgacatactgaggag-3' and antisense, 5'-tcacatagctcttctgtgtc-3'), *NECAB2* (sense, 5'-cgacagaaccacatcaaacc-3' and antisense, 5'-atcctccgtggcagatggaa-3'), *NECAB3* (sense, 5'-ccgcagagcagacaagaatg-3' and antisense, 5'-ccaggtgctctgagaagtag-3') were used for real-time PCR.

Antibodies. Rabbit antibodies against calnexin (Abcam, Cambridge, U.K.) and EEA1 (Cell Signaling Technology, Danvers, MA) were used for immunostaining.

Supplementary Figures.

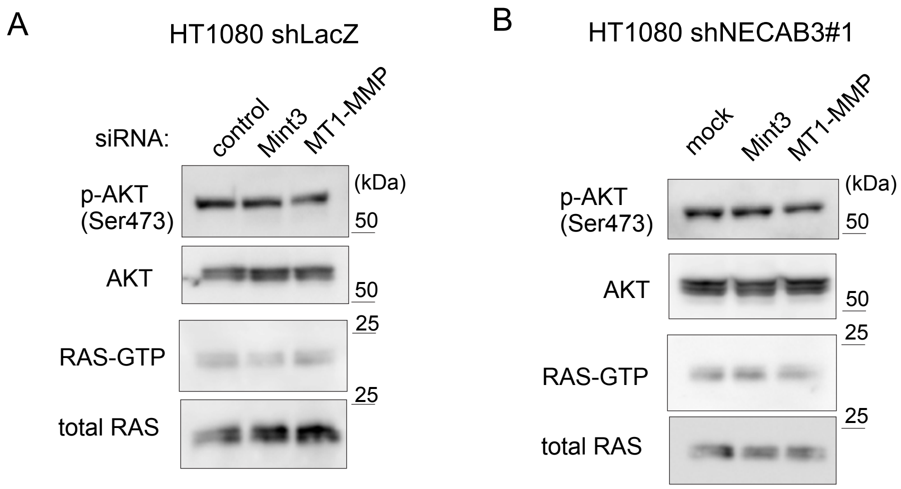

Figure S1. Knockdown or overexpression of MT1-MMP and Mint3 did not affect AKT phosphorylation and Rac activation. (A-B) Immunoblotting of phospho-AKT (Ser473), AKT, active Ras (Ras-GTP), and total Ras in whole cell lysates from control (HT1080shLacZ, **A**) or NECAB3-depleted cells (HT1080shNECAB3#1, **B**) transfected with siRNAs against MT1-MMP, Mint3, or control sequences.

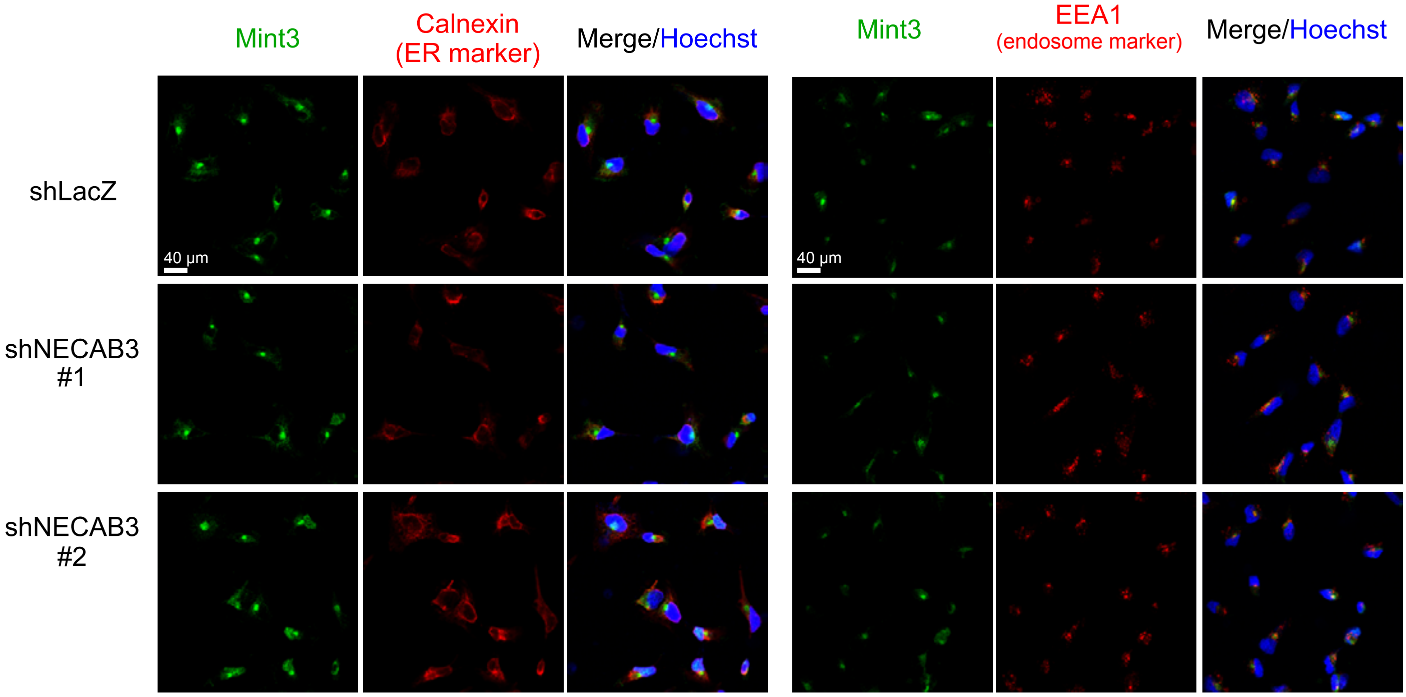

Figure S2. Immunostaining of Mint3, calnexin, and EEA1 in NECAB3-depleted HT1080 cells.

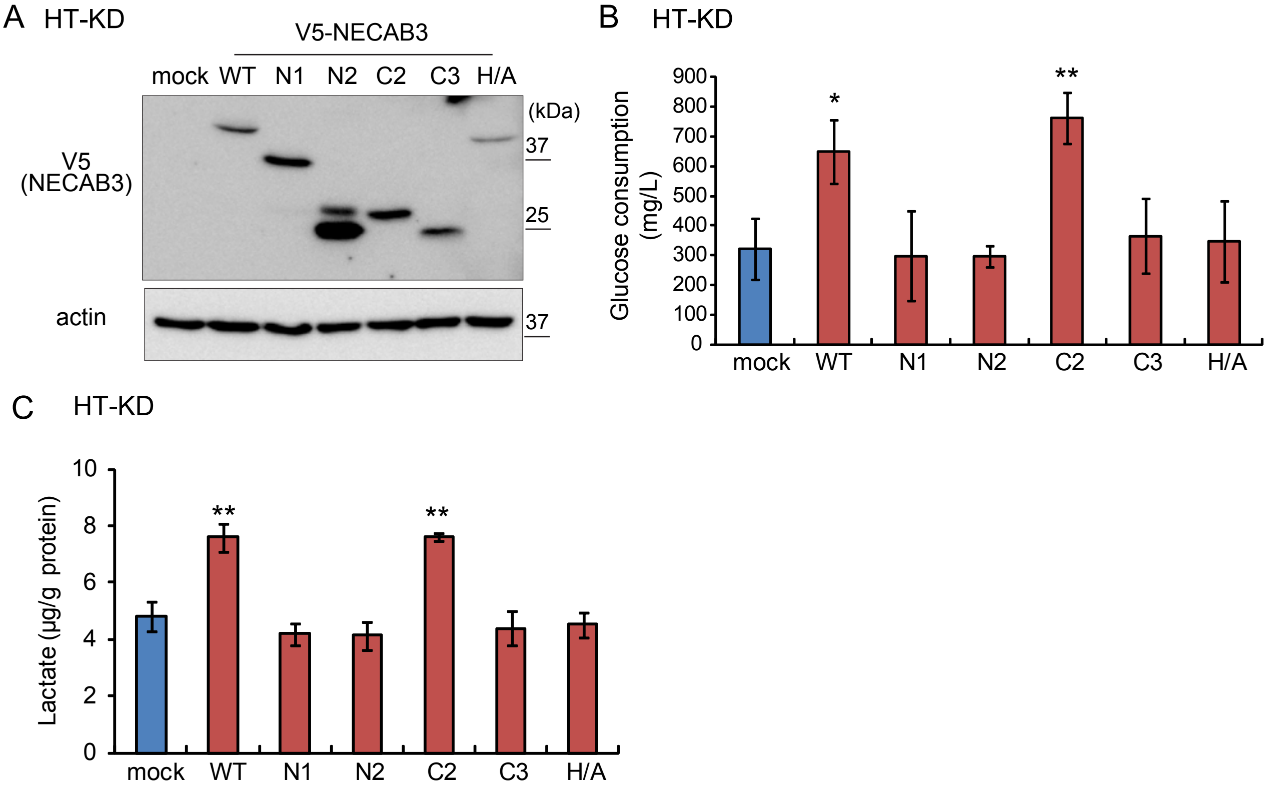

Figure S3. Wild-type and C2 mutant of NECAB3 restores glycolysis during normoxia in NECAB3-depleted HT1080 cells.

(**A**) Expression of V5-tagged NECAB3 and its mutants in NECAB3-depleted HT1080 cells.

(**B**, **C**) Glucose consumption (B) and lactate production (C) in NECAB3-depleted HT1080 cells expressing NECAB3 derivatives. In B and C, error bars indicate s.d. (n = 3), and data were analysed by t-test. *, $p < 0.05$ and **, $p < 0.01$.

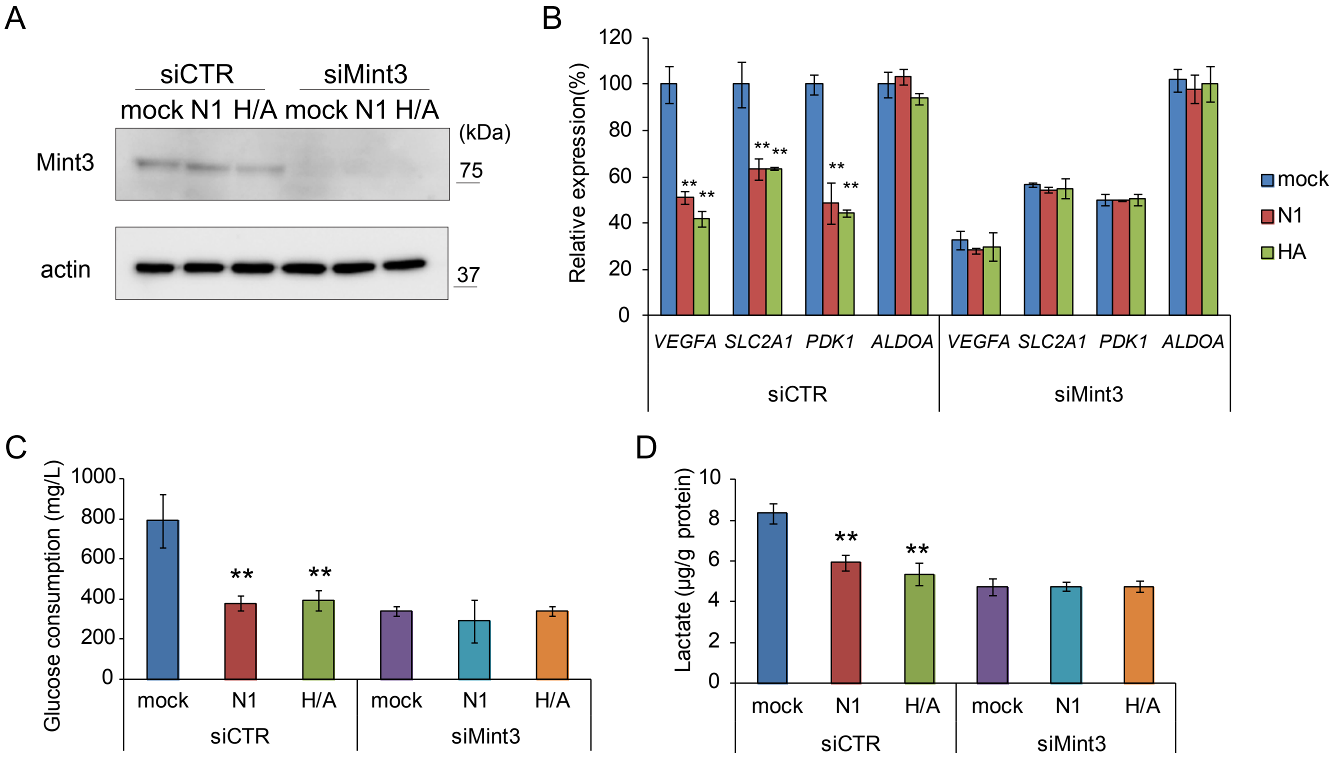

Figure S4. Dominant-negative effects of NECAB3 mutants require Mint3.

(A) Immunoblotting of Mint3 and actin in whole lysates from HT1080 cells expressing mock, NECAB3 N1, or H/A mutants and transfected with control or Mint3 siRNA.

(B-D) Gene expression (**B**), glucose consumption (**C**), and lactate production (**D**) in HT1080 cells expressing mock or NECAB3 dominant negative mutants and transfected with control or Min3 siRNA.

In **B**-D, error bars represent s.d. (n = 3), and data were compared pairwise by t-test. **, $p < 0.01$.

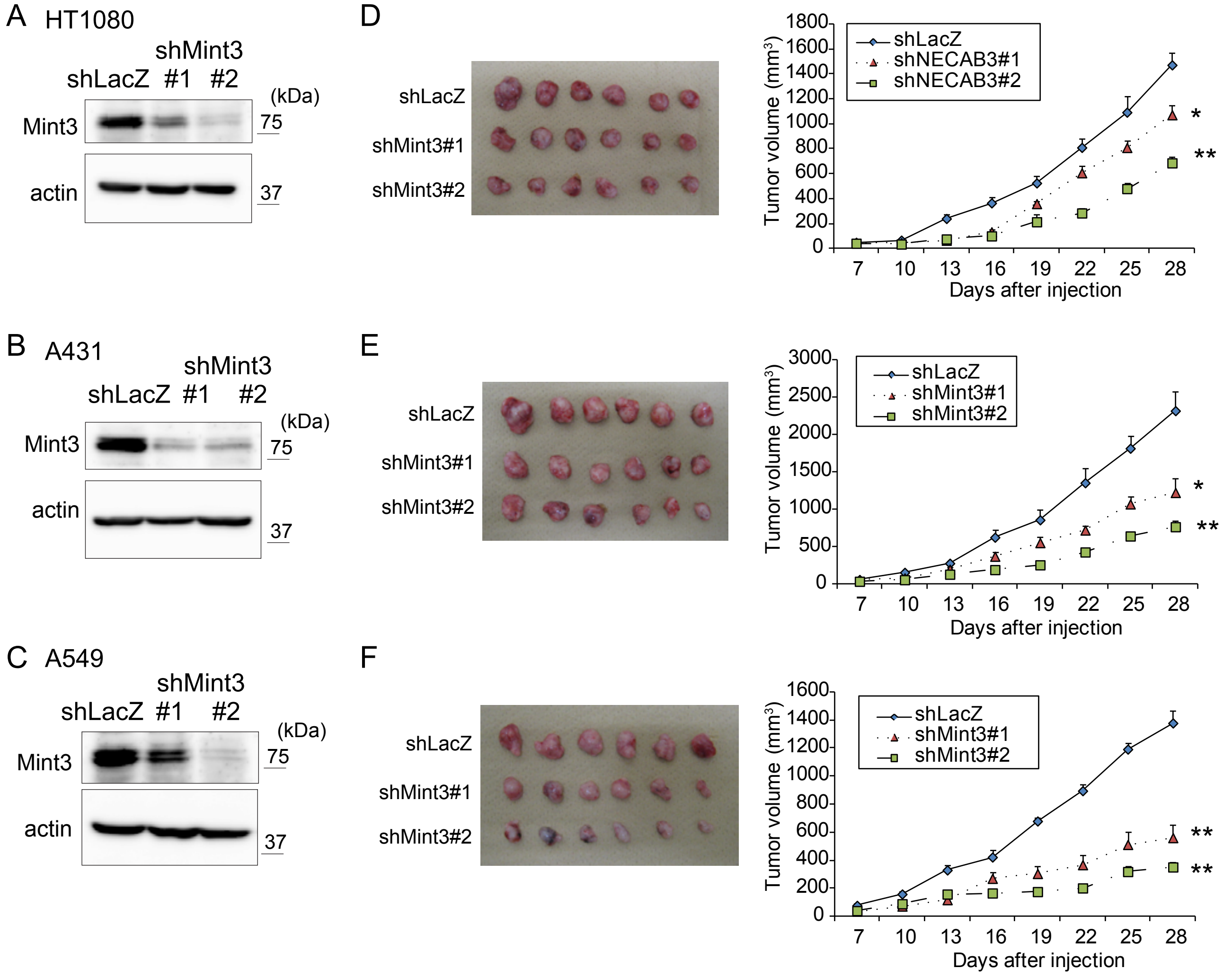

Figure S5. Mint3 depletion attenuates growth of cancer cells implanted in vivo.

(A-C) Immunoblotting of Mint3 and actin in whole cell lysates from control (shLacZ) or Mint3-depleted (shMint3) HT1080 (**A**), A431 (**B**), and A549 cells (**C**).

(D-F) Growth of Mint3-depleted HT1080 (**D**), A431 (**E**), and A549 cells (**F**) implanted into immunodeficient mice. Photos show tumours at day 28 after injection. Error bars indicate s.e.m. ($n = 6$), and data were analysed by Mann-Whitney U-test. *, $p < 0.05$ and **, $p < 0.01$.

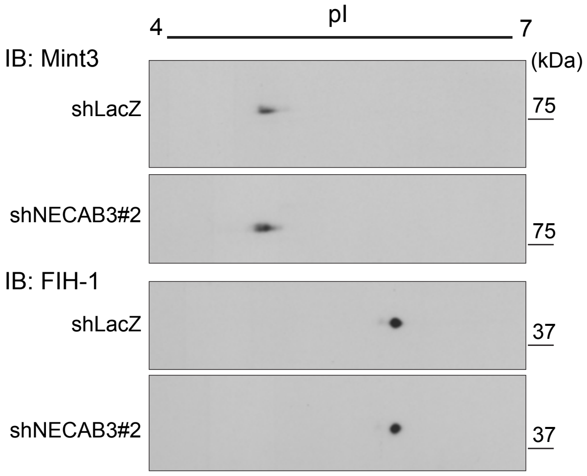

Figure S6. NECAB3 depletion does not affect phosphorylation of Mint3.

The acidity of Mint3 and FIH-1 in control (shLacZ) or NECAB3-depleted HT1080 cells was analysed by 2D electrophoresis and immunoblotting.

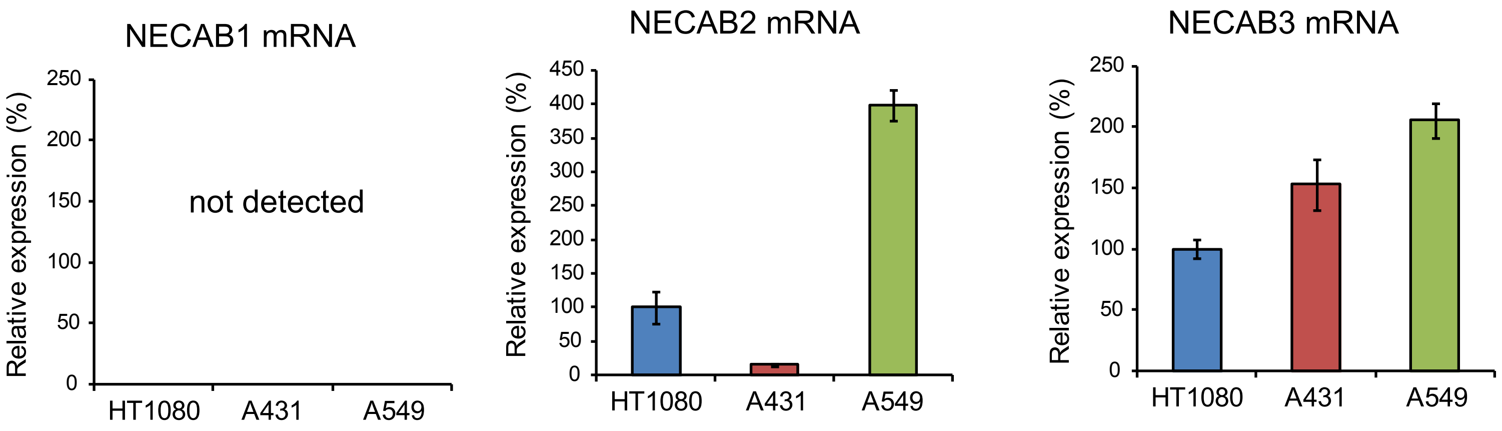

Figure S7. Expression of NECAB genes in cancer cells.

Expression of NECAB1, NECAB2, and NECAB3 was analysed by real-time PCR in HT1080, A431, and A549 cells. NECAB1 expression was not detectable in all cells.

gene name	reference sequence	start	stop
NECAB3	NM_031231.2	336	972
		546	1019
APLP2	NM_001642.1	2124	2581
		2127	3191
		2139	3190
		2142	3248
		2160	3084
		2166	2579
		2184	3238
		2184	3188
		2184	3252
		2184	3238
		2208	3558
		2235	3080
BTBD15	NM_014155.3	51	1602
		360	1599
		687	1576
		1032	1485
ESE-1	U73844.1	645	1229
HSD17B1	NM_000413.1	-385	694
		-376	318
		-373	699
SOX13	NM_005686.2	903	1594
SOX7	NM_031439.2	-70	860
		-58	871
ZNF143	NM_003442.4	330	1448

Table S1. Prey fragments identified from yeast two-hybrid screens against Mint3.
